# Monthly Summary

**Published:** 1876-04

**Authors:** 


### MONTHLY SUMMARY.

---

*Radical Cure of Salivary Fistula.*—J. H., aged ten years, suffered from an abscess beneath the right ear, at the lower margin of the parotid gland, and applied to a physician for treatment. A free incision was made, pus evacuated, and the patient relieved. The incision did not heal completely, leaving a fistulous opening at the most dependent portion of what had formerly been an abscess, and an almost constant discharge was the result.

This condition continued for seven years. Patient applied to me for treatment one month ago. On examination I found a salivary fistula, opening externally and immediately posterior to the angle of the inferior maxilla. Steno's duct had been divided at a point corresponding with its exit from the parotid gland, and there had been formed an artificial channel leading from the gland downward to the lowest part of the incision, as shown by the cicatrix.

My first effort was to explore the duct of Steno. I passed my probe into the duct to within about three-fourths of an inch of the fistula, where I met an obstruction. Beyond this point the duct had been obliterated. I then cut down, from without at the point of the probe. I then passed a needle, armed with a strong silk thread, from the opening just made in the cheek to a point about a line above the fistula, penetrated the artificial channel above the fistula, and, turning the point of the needle downward, brought it out at the fistula, allowing the thread to remain as a seton. In this way I hoped to make an artificial duct from the fistula to the artificial opening just made. After thirty-six hours I removed the seton and passed a small-eye probe along the track of the seton from the artificial opening to the fistula, then armed the probe with a silk thread, having a knot in one end, drew the probe backward and out, leaving one end of the thread on the outside of the cheek at the artificial opening, after having buried the knot at the other end in the artificial channel above the fistula, and then closed the fistula with a suture externally. After the artificial duct from the fistula to the artificial opening in the cheek had been sufficiently established to permit the saliva to flow out freely upon the cheek at the artificial opening, I was ready for the next step in the operation. I then moved the fistula forward on the cheek and closed the fistula behind the angle of the jaw. I wanted to know certainly that an artificial duct had been established from the fistula to the opening made in the cheek before I proceeded further, else I should have brought the thread forward through the natural duct and finished the operation.

I next passed my probe backward through the duct from the inside of the mouth to the artificial opening in the cheek, armed the probe with a silk thread, and again withdrew it, drawing the thread through the natural duct, leaving one end of the thread on the inside of the cheek suspended from the natural opening of Steno's duct, opposite the second upper molar, while a knot in the other end of the thread retained that end in the artificial opening. I then closed the opening in the cheek with plaster, and the saliva has since been flowing out at the natural opening. Both external openings have healed. The saliva readily followed the thread, the thread acting as a conductor, hence I preferred it to silver wire. The cure is radical.—*Clinical Record.*

---

*Morphia in Neuralgia.*—Mr Spencer Thompson writes to the *Lancet* on the subjectof neuralgia :—

Even phosphorus, we know, will, after a time, lose its power in some obstinate cases of neuralgia, at all events its power of giving rapid relief; and then it is that the invaluable hypodermic administration of morphia comes to our aid. This remedy, and its mode of administration, are too well known to require comment here; but it is far from being as generally employed as it ought to be. This, perhaps is due to various causes, but of these I believe the principal are, the means of administration not being always readily available, and the objection of patients to the pain consequent upon the use of coarsely constructed instruments. The first of these objections I have endeavored to meet by the use of a very portable hypodermic apparatus, enclosed in a metallic case, with ample supply of needles, and the great desideratum, an always moist and efficient piston ; and by always carrying a supply of gelatine discs. The second objection is met by the use of very fine steel needles only. The discs, which contain one-sixth of morphia in each, are a very safe and efficient dose for most cases, although in some it may be well to begin with the less amount, and in many it may be advisable to increase the dose considerably, half a grain, or even double that amount. I may here give it as the result of a very large experience in the hypodermic administration of morphia, that concentrated solutions are the reverse of advantageous. In the first place, they are not so safe as the more dilute; and, in the second, they do not act so quickly and agreeably. The usual strength I employ is one grain of hydrochlorate of morphia in forty minims of water, rarely in thirty. The slight increase of bulk is of no consequence, and in administrations I can count by the thousand, I have never seen the slightest bad consequence, in the way of abscess or otherwise, result to the patient. —*Med. and Surg. Reporter.*

*A New Solvent for Salicylic Acid.*—M. Rozsyay states, in a communication to the *Pharm. Centralhalle,* that he has found in the sulphite of soda an agent which increases the solubility of salicylic acid, as well as its antiseptic properties. One part of salicylic acid, with the addition of two parts of sulphite of soda, dissolves in fifty parts of cold water, and forms a clear solution, which is not in the least degree irritating to an open wound. As a disinfectant he uses one part of the acid with from one to two parts of the sulphite of soda in from fifty to a hundred parts of water.—*Med. Chir. Centralbl.*, 1874, 26.—*Rundschau,* Aug., 1875.

---

*How to Prevent Chloroform Asphyxia.*—Dr. J. Fleiberg. (*Berl. Klin. Wochenschr.*

His plan is to dislocate the inferior maxillary bone forward whenever any asphyxial symptoms make their appearance. The best way of producing this dislocation is to stand behind the reclining patient, put both thumbs behind the symphysis and the index fingers on the posterior edges of the rami of the bone, then grasp the maxilla firmly and drag it directly forward. If the patient be under the influence of the anæsthetic—and then only it is necessary to resort to this procedure—the condyle will move forward with a perceptible motion and the entire bone is displaced. As soon as this is done the patient takes a long breath and respiration proceeds without any further difficulty as long as the parts remain in the same position. The author has employed this procedure in more than a thousand instances and has never failed to achieve the desired purpose namely, the use of chloroform without any unpleasant complications. He believes that the root of the tongue and the epiglottis are dragged forward and thereby the occlusion of the larynx and the consequent asphyxia which is due to the weight of the tongue, are obviated.

The same method has been in constant use by Esmarch since 1866 ; and Langenbeck employed it constantly during the late Franco-German war, and never lost a case from the effects of chloroform.—*Chicago Med. Jour. and Examiner,* Jan., 1876.

*To Retard the Setting Gypsum.*—It has been found that ground plaster sets most speedily when it contains twenty per cent. of water. An excess of water, however, causes the solidification to be considerably retarded. There are several other substances which, when added, conduce to the same result; such as gelatine, glycerine, gum, common salt, marsh mallow, etc. Carbonate of lime is said also to be an efficient means for preventing the rapid setting. It is often useful when employing plaster-of-paris to prolong the time required for it to harden, and it is convenient to know how to effect that result. To the foregoing it may be appropriate to add, that Mr. Gaudin, of Paris' has lately patented a method of treating old plaster that has been broken up and condemned, which fits it again for renewed use, and is not inferior to its original calcined condition. After it has been roasted the second time, and rendered anhydrous, old plaster sets too quickly for common purposes. By calcining the rubbish and mixing it with some of the saline solutions instead of pure water, this objection is prevented. Alkaline solutions are best, and of these a solution of carbonate of soda is preferred because it is the cheapest. When treated in this manner, such plaster, will harden by the end of a couple of hours, and possess all the useful properties of fresh gypsum,—*Druggists' Circular.*

---

*Eczema Produced by Tincture of Arnica.*—Dr. Whittaker has (*Ohio Clinic*) lately encountered several cases illustrating the evils of the local application of tincture of arnica. One young man with orchitis applied it, and came to Dr. Whittaker with an extensive and profound eczema, which lasted for three week's Another man, having let a ten-pin ball fall upon his toes, applied it to relieve the pain, and had a violent eczema, which was not cured for two weeks. At the society at which this was read, Dr. Longworth mentioned the fact as something peculiar about this drug; the majority of persons may use it with impunity, but it will now and then act as a virulent poison, producing an eczema not limited to the seat of its application, but which becomes universal, and is often very obstinate to treatment.—*Atlanta Med. and Surg.*

*A New Pair of Siamese Twins.*—At a recent meeting of the New York Pathological Society, Dr. John E. Blake, presented a pair of infants joined after the fashion of the Siamese twins. The specimen was merely loaned to him by a gentleman who had purchased it for the Anatomical Museum at Sweden. The twins (females) were born in Tobasco, a town in Mexico, are reported to have lived nine days, and to have weighed 15 pounds. The mother was an Indian and the father a mulatto. One twin showed the characteristics of the former race and the other of the latter. In this connection it is well to state that the *Student's Journal and Hospital Gazette* (London, Feb. 12) reports a similar case. "The wife of a carpenter residing in Hastings was delivered of twins (girls,) who were united by a cord or ligament of flesh drawing both breasts together." The infants, who were well developed and possessed pretty features, did not survive their birth. The case has attracted some attention among medical men in that place, and photographs of the twins have been taken.—*Med. Record.*

---

*Death from Ether.*—Dr. Finnell presented the larynx of a patient who died at the Homœopathic Hospital while he was undergoing an operation for the removal of necrosed bone from the superior maxilla The larynx was œdematous. The patient was the subject of necrosis of the superior maxilla, and an operation was deemed advisable. Ether was administered, and an incision made through the lip, extending in a semicircular direction over the superior maxilla. At this stage of the operation, the patient became cyanotic, and soon died. The patient was immediately held up by his heels, with a view of clearing the trachea of blood, as it was suspected of being the cause of the difficulty, but this measure proved of no avail. The time which elapsed between the commencement of the administration of the ether and the death of the patient was ten minutes. Two and a quarter ounces of ether was the amount used, and was the purified ether manufactured by Dr. Squibb.

*Autopsy.*—No blood could be discovered in the trachea. The heart weighed six ounces, and was fatty. No other lesions were found anywhere. A strong odor of ether emanated from the brain.—*Proceedings of N. Y. Pathological Society.*

*Solvents for Rubber.*—The proper solvents for caoutchouc are ether (free from alcohol,) chloroform, bisulphide of carbon, coal naphtha, and rectified oil of turpentine. By long boiling in water, rubber softens, swells, and becomes more soluble in its peculiar menstrua; but when exposed to the air, it speedily resumes its pristine consistence and volume. Industrially, the etherial solution of caoutchouc is useless, because it contains hardly more than a trace of that substance. Oil of turpentine dissolves caoutchouc only when the oil is very pure, and with the application of heat; the ordinary oil of turpentine of commerce causes india-rubber to swell rather than to become dissolved. In order to prevent the viscosity of the india-rubber when evaporated from its solution, one part of caoutchouc is worked up with two parts of turpentine into a thin paste, to which is added half part of a hot concentrated solution of sulphuret of potassium in water; the yellow liquid formed leaves the caoutchouc perfectly elastic and without any viscosity. The solutions of caoutchouc in coal tar naphtha and benzoline are most suited to unite pieces of caoutchouc, but the odor of solvents is perceptible for a long time. As chloroform is too expensive for common use, sulphide of carbon is the most usual and also the best, solvent for caoutchouc. This solution, owing to the volatility of the menstruum, soon dries, leaving the latter in its natural state. When alcohol is mixed with sulphide of carbon, the latter does not any longer dissolve the caoutchouc, but simply softens it, and renders it capable of being more vulcanized. Alcohol also precipitates solutions of caoutchouc. When caoutchouc is treated with hot naphtha distilled from native petroleum or coal tar, it swells to thirty times its former bulk; and if then triturated with a pestle and pressed through a sieve, it affords a homogeneous varnish, the same that is used in preparing the patent waterproof cloth of Mackintosh. Caoutchouc dissolves in the fixed oils, such as linseed oil, but the varnish has not the property of becoming concrete on exposure to the air. Caoutchouc melts at a heat of about 256° or 260°; after it has been melted, it does not solidify on cooling, but forms a sticky mass which does not become solid even when exposed to the air for months. Owing to this property, it furnishes a valuable material for the lubrication of stopcocks and joints intended to remain air-tight and yet be movable.—*Jour. of Applied Science.*

*A Third Dentition at the Age of Seventy-Three*—M. Echerac relates in the *Gazette des Hospitaux,* the remarkable case of an old gentleman, aged seventy-three, who, after the manifestations of nervous symptoms for some time, and an abundant salivation, exhibited in his upper jaw, which had long been dismantled of teeth, some fine ones projecting about two millimetres beyond the edge of the gums. They were six in number—four incisors, one canine, and one small molar. These teeth were neither very white nor very strong, but formed an excellent substitute for those lost. Van Helmont relates a precisely similar case occurring at the same age.

---

*How to Remove Stains from Steel Instruments.*—The very best way to clean a stained steel knife is to cut a solid potato in two, dip one of the pieces in brick-dust such as is usually used in knife-cleaning, and rub the blade with it.

---

*Toothache.*—M. J. Eley, M. D., in Medical and Surgical Reporter says, you will do something for the afflicted and possible preserve the morals of many, by saying to your readers that acetate of lead, applied on moistened cotton will relieve most cases of this, Burns' "hell of diseases." As fast as it dissolves spit out, and re-apply. I think it fails less than any other remedy I have prescribed.

---

*Treatment of Diphtheria.*—Dr. J. S. Benson writes to the Canada *Lancet:*—I take the liberty of placing before your readers the treatment which has proved in my hands not only very efficacious, but almost a complete specific. For the last twelve or thirteen years we have have had unequaled opportunities of watching the disease in all its stages, from the mere fibriny whiteness of the palate and tonsils to the complete obliteration of the palatine arch by false membrane internally, and the enormously enlarged sub-maxillary and parotid glands externally, which sometimes suppurated; death resulting in some cases from the mechanical obstruction to respiration, and in others from pure blood-poisoning as manifested through the nervous system; the local symptoms in the throat having to a great extent disappeared, leaving both food and air passages perfectly free and open.

When called to a case of diphtheria, I first of all clear out the bowels by a dose of calomel, followed in a few hours by a little senna or Rochelle salts. If there is much heat of skin, as will be shown by the thermometer, a warm bath will give very great relief; and after the bowels have been freely acted upon I commence with the chlorine water. To patients from ten to twelve years and upward I give one tablespoonful; from six to twelve, one dessert-spoonful, and under six one teaspoonful, every three hours. In all cases it should be given without any addition of water. A towel saturated with cold spring-water is to be applied to the throat and neck, the cold renewed as the towel becomes warm. The patient is to have small pieces of ice on a plate by the bedside, a piece of which is to be kept constantly in the mouth. The diet should be nutritive; beef tea, milk, eggs, and such like; but no stimulant is required, unless the prostration should be alarming. In some cases which are mild, and occur in young children, a mixture containing tincture of iron, chlorate of potash, and dilute hydrochloric acid, may suffice to cure, but in no case can the same dependence be placed in it as chlorine water.

Notwithstanding the opinions of Greenhow, Brettonneau, and others of equal celebrity, as to the efficacy of caustics and gargles as topical agents, I must humbly beg leave to differ entirely with them, and disapprove in *toto* of such proceedings. I most assuredly believe them not only to be of no value, but in many cases positively injurious. If we look upon diphtheria as a blood-poison, which it undoubtedly is, we must, if possible, find an antidote and administer it. I believe we have it in the above mixture. Can we expect to cure it by a little nitrate of silver locally applied, and giving a little tonic medicine? Here is a disease, a poison in the blood, manifesting itself and increasing so rapidly in a part the free passage through which it is indispensable to life, which, if not arrested, will in a few hours place the patient beyond the reach of human aid. It is not a disease which we can allow, as some fevers, to run a definite course, merely supporting the strength and guiding it to a certain turning point. Such would, I think, be a dangerous experiment in diphtheria.

*To the Editor of the American Journal of Dental Science:*

In the May number of the Journal, handed me a few days ago, I read with much surprise and regret the remarkable attacks which you have chosen to make upon the Maryland Dental College.

It is exceedingly unpleasant at all times to be dragged into a controversy, which must naturally lead to crimination and recrimination; but for two Colleges in the same city, to get into contention in print, is especially to be deplored and deprecated,—to say nothing of the evil influence it must necessarily have upon the standing of the Journal over which as Editor you have the honor to preside; still, you have left me no alternative but to defend the College with which I am connected, and the responsibility therefore rests wholly with yourself.

Why you chose to publish from an anonymous source, statements in regard to the Maryland Dental College, and its number of matriculants, *which are utterly false*, when you could so easily have gotten at the truth of the matter, *I shall not discuss;* but simply ask in justice to the College of which I have the honor to be Dean, that you publish the address and names of *the eleven*—(11) gentlemen who were matriculants, and in actual attendance upon the school.

1. F. F. Drew, D. D. S., No. 160 W. Madison St., Balt.
2. Wm. H. Gingrich, D. D. S., Reistertown, Maryland.
3. Wm. H. Law, D. D. S., Hayardville, Conn.
4. F. W. Sheild, D. D. S, No. 119 N. Charles St., Balt.

---

5. J. Anson Bates, M. D., No. 169 N. Charles St., Balt.
6. Robert B. Cromer, No. 305 Hollins St., Baltimore.
7. Ase. Petit le Brune, No. 128 W. Madison St., Balt.
8. Cyrus M. Gingrich, Reistertown, Maryland.
9. W. C. Greene, M. D., Wilkesboro, N. C.
10. Richard M. Johnson, Louisburg, N. C.
11. Frederick A. Walls, No. 181 E. Monument St., Balt.

In regard to the suggestion from this same *anonymous source*, that the College "would seem to need looking after by its Board of Regents," I will in justice both to our Regency and the College itself, ask the publication of the following papers which would seem to demonstrate the fact most clearly, that the Regents have not been derelict in their duty.

In conferring the degrees, Cornelius T. Hurlbut, D. D. S., of Springfield, Mass., addressed the graduates as follows:

*Gentlemen:* It affords me great satisfaction to have the pleasure this evening to confer upon you a degree for which you have so nobly striven and honorably won.

It has been the custom of all Dental Colleges in the past, to grant a like degree when the student has passed a satisfactory examination before several Professors comprising the Faculty. The duty of conferring these degrees is wholly confided to the Board of Regents, and men that have had no agency in establishing the Maryland Dental College, yet they have cheerfully consented to assume the duties of their position from a willingness to second the efforts of the gentlemen of the Faculty in building up a school for promoting dental education and elevating the standard of professional attainments. That we might not be remiss in our duty, we have required of you a personal examination, which was highly satisfactory to us and creditable to you, and we say, without fear of contradiction, an examination that was never required of any dental student either in this or any other country. The Regents present not only consider you properly prepared to commence the practice of your chosen profession, but satisfied that the Faculty are eminently qualified for the positions they occupy in the College.

The Regents intended as last year to bestow "Class Honors" upon one or more members of this class, but after the final and thorough examination, we have concluded that it will not be possible to properly discriminate between the members of this class in regard to the efficiencies of scholarship and workmanship. In closing he made the following charge:

As a member of the dental profession you promise to maintain its honors and to labor earnestly to extend its sphere of usefulness, and avoid everything in language and conduct calculated to dishonor the profession. Do you this solemnly promise?

To this an affirmative response was given, and the graduates received their official parchments.

---

Letter written by the members of the Board of Regents, who were present at our last annual commencement and examinations, to the members of the Board who were absent:

### MARYLAND DENTAL COLLEGE.

BALTIMORE, APRIL, 1875.

*Gentlemen of the Board of Regents of the Maryland Dental College:*

Having attended the closing of the Second Session of the Maryland Dental College, we choose this method to assure you of the prosperous condition and favorable prospects of this institution, in which as honored Regents, you doubtless feel a deep interest.

Our frequent intercourse with the gentlemen composing the Faculty has fully satisfied us of the high capabilities which they possess for the successful discharge of their arduous duties; and you may rest assured students in this College will enjoy the advantages of a most thorough and conscientious course of instruction and preparation for practice in dentistry.

The devotion, zeal and energy of the Faculty have been remarkably displayed in the successful and permanent establishment of a dental school of high standing; and those who occupy the distinguished position of Regents therein will doubtless take great pleasure in using endeavors, according as they may have the power and opportunity, to contribute their influence to increase the efficiency of its management, and to extend the beneficence of its operations.

The gratification and encouragement which it would afford all concerned in the College to see a large attendance at the next annual examination, it is hoped will stimulate the members of the Board to endeavor to be present on that occasion.

Very respectfully,

R. B. Donaldson, *President.*
Geo. S. Fouke, *Vice President.*
R. Finley Hunt, *Secretary.*

This anonymous writer has also promised that he will hereafter bring to light other "invidious acts" of the Maryland Dental College; let him do so, but let them be stated over his own name, and not write under the signature of three little stars.

I hope next time he will be more accurate in his "well known" facts, and if *he too is not also a member of the Faculty of the Baltimore Dental College*, I shall be most agreeably disappointed.

The Editor of the Journal states, that *we* "do not desire the Baltimore College to be confounded with the one in question" (the Maryland Dental College.) *Has it ever occurred to him for a moment that such a confusion might be as objectionable to the one as to the other?*

R. B. Winder, Dean,
*Maryland Dental College.*

---

*A Protest Against, and Resignation of Dr. Thos. B. Gunning* from Board of Regents of the lately started Dental College, in which he dissolves all official connection with the same, and accuses the so-called Faculty of publishing a letter leading to injurious tendencies as regards the manner in which they propose to conduct their institution. Published in pamphlet form by Dr. Gunning.

Dr. Gunning makes use of the following language in regard to this point: "Admit it once, and every city, town or community that can muster a few ambitious dentists, is authorized to have its own Dental College, the requisite being only that the Faculty shall be endorsed by the clergymen, physicians and lawyers of their acquaintance."

This is certainly to the point, and the Doctor's eyes have evidently been opened, and he discovers that "all is not gold that glitters."—*American Journal Dental Science, Under Head of Bibliographical, May*, 1875.

*To the Editor American Journal of Dental Science:*

Dear Sir.—The general character of the system of Ethics adopted by our profession, and an ordinary observance thereof, should have rendered unnecessary the production and publication of the present paper. There should be no need for me to tell you what course of conduct towards members of the same profession is prescribed by this system of Ethics. This you were doubtless taught when receiving your dental education. My surprise was therefore great when I saw by the appearance of certain articles in the May number of the *American Journal of Dental Science*, that you as its Editor, had joined in an attack on the Maryland Dental College, and on the gentlemen engaged in its management. True, you made the publication of a pamphlet by Dr. Thos. B Gunning, of New York, (which you could not have read in a careful manner,) the occasion of your attack. With that pamphlet I do not propose to deal on the present occasion, but with your attack on the College, the motives therefor, and its justification, if any. In the first place then, Dr. Gunning's quarrel with the Maryland Dental College was not yours of necessity,—only when you made it so voluntarily. It is proper first to look for the justification, because the grounds which would justify this attack, or the want of them, must determine to a great extent the motives therefor. With this object in view it is natural to look at the constituted authorities of the College, and their official action, as well as the regime, both as regards principle and practice adopted by them. Let us take first what you are pleased to designate "the so-called Faculty" "of the lately started Dental College." The following are the names of the gentlemen who compose the Faculty of the Maryland Dental College, and the positions they respectively occupy:

*Faculty.*—SAMUEL H. WILLIAMS, D. D. S., Emeritus Prof. of the Institutes of Dentistry; BYRON F. COY, D. D. S., Prof. of Dental Surgery; HENRY H. KEECH, M. D., D. D. S., Prof. of Pathology and Therapeutics; M. WHILLDIN FOSTER, M. D., D. D. S., Prof. of Dental Mechanism and Metallurgy; EDWARD P. KEECH, M. D., D. D. S., Prof. of Clinical Dentistry and Materia Medica; RICHARD B. WINDER, M. D., D. D. S., Prof. of Physiology and Hygiene; SAMUEL M. FIELD, D. D. S., Prof. of Physics and Chemistry; L. McLANE TIFFANY, B. A., M. D., Prof. of Anatomy; A. P. GORE, D. D. S., Adjunct Prof. of Clinical Dentistry; C. T. BROCKETT, M. D., D. D. S., Demonstrator of Practical Dentistry; H. G. ULRICH, D. D. S., Demonstrator of Practical Dentistry; C. E. DUCK, D. D. S., Demonstrator of Practical Dentistry; B. W. BARTON, M. D., Demonstrator of Anatomy.

RICHARD B. WINDER, Dean of the Faculty,
*No.* 140 *Park Avenue, Baltimore, Md.*

These gentlemen are well known as occupying positions both socially and professionally among the highest in the community, and as a Faculty, certainly will not suffer by comparison with that of any other dental college. Your remark that "all is not gold that glitters," cannot therefore refer to the Faculty Has it reference then to the Board of Regents? This would seem possible, because Dr. Gunning's resignation and pamphlet, (which latter was made the occasion of your attack,) were based on the action of the Board of Regents. The following list will show who are Regents of the Maryland Dental College:

REGENTS.

Prof. Nathan R. Smith, M. D., Baltimore, Md.
Rev. Richard Fuller, D. D., Baltimore, Md.
Rev. L. F. Morgan, D. D., Baltimore, Md.
Chief Justice Geo. Wm. Brown, Baltimore, Md.
Col. Charles Marshall, Baltimore, Md.
Hon. Edwin H. Webster, Belair, Harford County, Md.

R. Finley Hunt, Dentist, Washington City.
B. F. Arrington, M. D., D. D. S., Raleigh, N. C.
J. G. Wayt, D. D. S., Richmond, Virginia.
James Johnston, M. D., Staunton, Virginia.
Cornelius S. Hurlbut, D. D. S., Springfield, Massachusetts.
George Shindler Fouke, Dentist, Westminster, Md.
George W. Neidick, D. D. S., Carlisle, Penn.
J. R. Walker, D. D. S., New Orleans, La.
J. C. Storey, M. D., D. D. S., Eutaw, Greene County, Ala.
W. W. H. Thackston, M. D., D. D. S., Farmville, Va.
Jas. F. Thompson, M. D., D. D. S., Fredericksburg, Va.
H. A. Lowrance, Dentist, Athens, Ga.
Rev. S. D. Noyes, D. D., Baltimore, Md.
Rev. Jas. D. McCabe, M. D., D. D. S., D. D., Balto. Md.
T. C. Edwards, D. D. S., Brownsville, Tennessee.
Rev. Myer Lewin, D. D., Port Tobacco, Md.
R. B. Donaldson, D. D. S., Washington, D. C.
George A. Mills, Dentist, Hartford, Conn.
Wm. T. Arrington, D. D. S., Memphis, Tenn.

As my name appears among them, I can only speak of my colleagues, and say that their high character and standing as citizens, and their extensive and honorable reputation as professional men, render this *seeming possibility impossible.* Then the justification for your attack must be looked for in the principles upon which the College is established, and the manner in which those principles are carried into practice. Let us see if there is anything wrong on these points. The Maryland Dental College is organized and conducted mainly as other dental colleges are, but some of its features are peculiar to that Institution. Part of these were adopted at the beginning,—the remainder March 2nd, 1875. One peculiar feature is the Board of Regents, a list of its members being given above. The Faculty desiring to give every assurance of the sincerity and earnestness of their purpose to elevate the standard of dental education, very wisely concluded that after leading and instructing the matriculants through the course, they would not sit in judgment on the result of their own labors, and accordingly they elected this Board of Regents and delegated to them the whole power of conferring degrees and of pronouncing upon the qualifications of candidates for graduation, their judgment to be based upon a personal examination of the candidates by the working members of the Board of Regents; that is, those belonging to the dental profession. The records and all concurrent evidence will show that the duties devolving respectively upon the Board of Regents and the Faculty, have thus far been faithfully performed. It was also decided March 2nd, 1875, that the Faculty shall determine by a preliminary examination, the fitness as to moral character and preparation as to previous education of all applicants for matriculation. Also, that knowledge, skill and proficiency in all the requisite branches and departments, coupled with a proper moral character, shall constitute the title to a degree and diploma without special reference to where, how, and in what number of years this knowledge, skill and proficiency had been acquired. Also, that previous to conferring the degrees on graduates, a pledge of honor shall be required of each one, that he will always act with professional honor and in accordance with professional Ethics. Is there anything in all this that "glitters and, though pretending, is not gold?" We who are connected with the College may be prejudiced, but we believe that our action has been founded upon plain, correct principles and sound common sense. If we are wrong, we are willing to be set right. If we are right, those who attack us do so without cause, and unless they can and do show where and how we are wrong, justice and courtesy alike demand that they should make proper amends. With regard to the motives which prompted your attack I decline at present to say anything.

Respectfully, yours, &c.,

R. FINLEY HUNT,

*Secretary Board Regents Maryland Dental College.*

